# Successful manual reduction for ureterosciatic hernia: A case report

**DOI:** 10.1016/j.ijscr.2019.03.036

**Published:** 2019-03-30

**Authors:** Jiro Kimura, Kentaro Yoshikawa, Takashi Sakamoto, Alan Kawarai Lefor, Tadao Kubota

**Affiliations:** a Department of Surgery, Tokyo Bay Urayasu Ichikawa Medical Center, Chiba, Japan; b Department of Surgery, Jichi Medical University, Tochigi, Japan

**Keywords:** Sciatic hernia, Manual reduction, Ureter

## Abstract

**Introduction:**

Sciatic hernias are the least common type of pelvic floor hernias. The purpose of this study was to present a novel technique for manual reduction and to conduct a systematic review of previous reports of sciatic hernias to characterize them and review the outcomes.

**Presentation of case:**

An 86-year-old female presented with left-sided lumbar pain. She had a past medical history of rheumatoid arthritis and was treated with prednisolone and methotrexate. Her left abdomen and left lumbar area were tender. An unenhanced abdominal computed tomography scan revealed invagination of the left ureter into the left sciatic foramen and a dilated left proximal ureter and renal pelvis. Ultrasonography showed an invaginated left ureter viewing from the left buttock. She was diagnosed with a sciatic hernia. Ultrasound-guided manual transvaginal reduction was performed. Post-procedure unenhanced abdominal computed tomography scan confirmed reduction of the ureter. After 10-months of follow-up, there is no evidence of recurrence.

**Discussion:**

Previous reports of patients with sciatic hernia were identified. Clinical data associated with the hernia, reduction technique and clinical outcomes were collected for 72 patients. Open reduction was performed in 24 patients. A ureteral stent was placed in eight patients when the hernia contained the ureter. Four postoperative complications including one death were reported in adults. There were no reports of closed manual reduction.

**Conclusion:**

A sciatic hernia in women may be manually reduced without surgery. Further reviews of this rare entity are needed to determine the best management strategy.

## Highlights

• Manual reduction was successfully performed for a patient with sciatic hernia.
• There were no report of closed manual reduction previously.
• A sciatic hernia in women may be manually reduced without surgery.

## 1 Introduction

Sciatic hernia is the rarest type of pelvic floor hernias, which includes obturator, perineal, and sciatic hernias. Sciatic hernias are characterized by the hernia contents entering the greater or lesser sciatic foramen. The greater sciatic foramen is subdivided by the piriformis muscle and atrophy of the piriformis muscle may be one cause of sciatic hernia. Sciatic hernia was first described by Papen in 1750 and observed and recorded by Verdier in 1753 [1].

The purpose of this study was to present a novel technique for manual reduction and to review published reports of sciatic hernias to summarize the experience to date in the management and outcomes of this entity. This work has been reported in line with the SCARE criteria [2].

## 2 Presentation of case

**Fig. 1:**
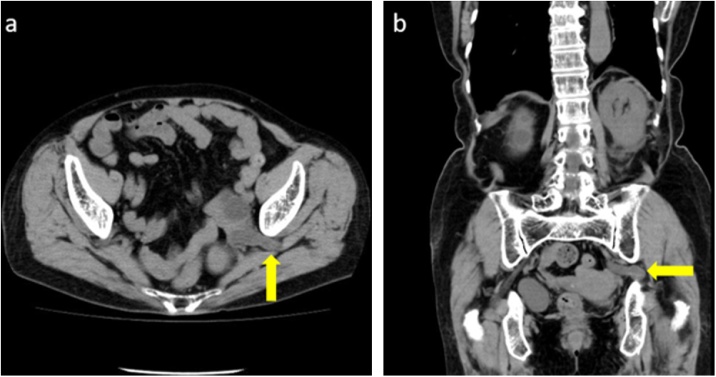
Unenhanced abdominal computed tomography scan revealed invagination of the left ureter into the left sciatic foramen (arrow). **a**. axial view, **b**. coronal view.

**Fig. 2:**
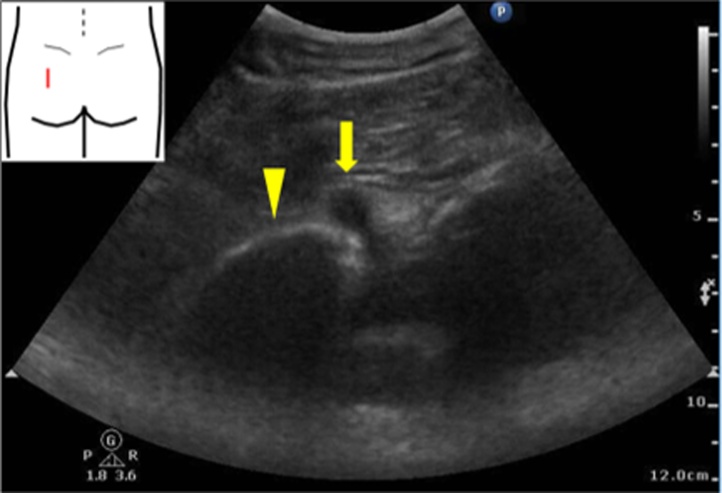
Ultrasonographic imaging shows an invaginated left ureter (arrow) and the ilium (arrowhead) when the probe was placed on the left buttock.

**Fig. 3:**
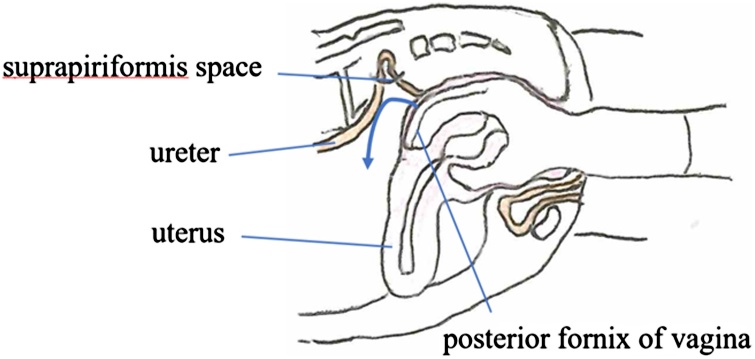
Ultrasound-guided manual transvaginal reduction was performed. Tension was placed on the ureter and nearby retroperitoneum by the right index and middle finger of the operator.

**Fig. 4:**
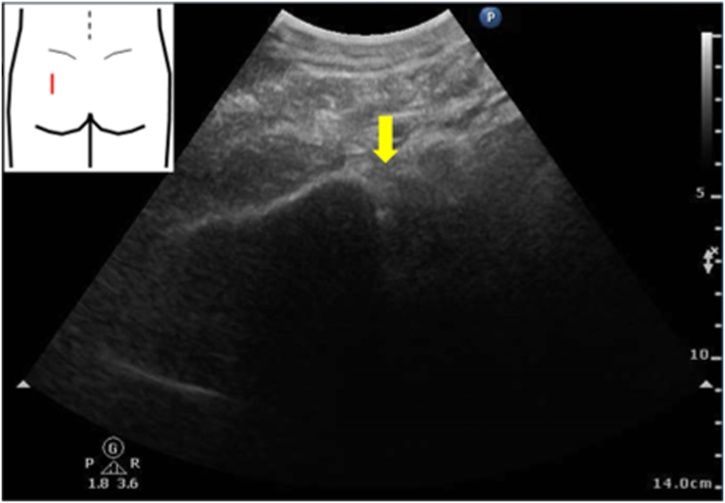
The ultrasound probe was placed on the left buttock of the patient during the procedure and the invaginated ureter was reduced (arrow).

**Fig. 5:**
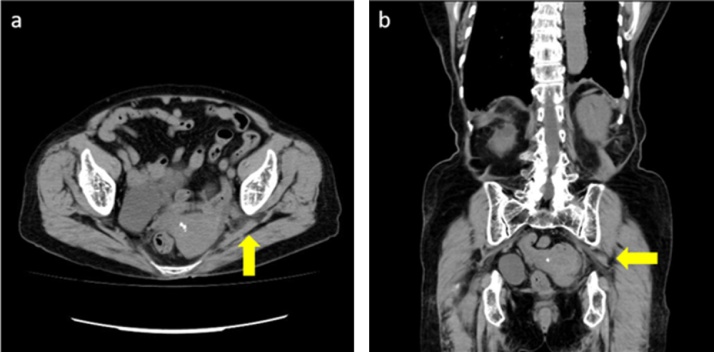
Post-procedure unenhanced abdominal computed tomography scan confirmed reduction of the left ureter (arrow). **a**. axial view, **b**. coronal view.

An 86-year-old female presented with left-sided lumbar pain. She had a past medical history of rheumatoid arthritis and was treated with prednisolone and methotrexate. On physical examination, her left abdomen and left lumbar area were tender. Laboratory examination showed no abnormalities. An unenhanced abdominal computed tomography (CT) scan revealed invagination of the left ureter into the left sciatic foramen and a dilated left proximal ureter and renal pelvis (Fig. 1). Ultrasonography showed an invaginated left ureter when the probe was placed on the left buttock (Fig. 2). The hernia orifice was 10 mm in diameter. She was diagnosed with a sciatic hernia. On the second hospital day, her symptoms continued and ultrasound-guided manual transvaginal reduction was performed. The patient was placed in the prone position in bed. The entire hand of the examiner was inserted into the vagina. Tension was put on the ureter along with nearby retroperitoneal tissue by the right index and middle finger of the examiner (Fig. 3). The ultrasound probe was placed on the left buttock of the patient. The invaginated ureter was then reduced (Fig. 4). Post-procedure unenhanced abdominal CT scan confirmed reduction of the ureter (Fig. 5). The post-reduction clinical course was uneventful, and she was discharged one day after the procedure. After 10-months of follow-up, there is no evidence of recurrence.

## 3 Discussion

A search of English-language abstracts in PubMed and Igakuchuo-Zasshi through 2017, with keywords of “sciatic hernia” or “ureterosciatic hernia” revealed a total of 71 patients with sciatic hernias [1,[3], [4], [5], [6], [7], [8], [9], [10], [11], [12], [13], [14], [15], [16], [17], [18], [19], [20], [21], [22], [23], [24], [25], [26], [27], [28], [29], [30], [31], [32], [33], [34], [35], [36], [37], [38], [39], [40], [41], [42], [43], [44], [45], [46], [47], [48], [49], [50], [51], [52], [53], [54], [55], [56], [57], [58], [59], [60], [61], [62], [63], [64], [65], [66], [67], [68], [69], [70]]. Of 72 patients with a sciatic hernia including the present patient, for whom comprehensive data were found, there were 61 adults (age 29–93 years) (Table 1) and 11 children (age 2–660 days) (Table 2).

**Table 1:** Reports of adult patients with a sciatic hernia.

No.	Author	Year	Age	M/F	BMI (kg/m^2^)	L/R	Hernia contents	Treatment	Follow up (months)	Complications
1	Summers [1]	1921	35	M	none	R	myxoma	Observation	NA	NA
2	Lindbom [3]	1946	54	F	none	L	left ureter	open, resection of the left ureter	NA	none
3	Lawson [4]	1948	39	M	none	R	small bowel	open, reduction of small bowel	1	none
4	Beck [6]	1952	66	F	none	L	left ureter	Open	NA	heart failure
5	Kerry [9]	1964	57	F	none	L	retroperitoneal lipoma	open and gluteal incision	none	none
6	Sadek [10]	1969	33	F	none	L	small bowel	open, resection of neurofibroma, 10 days later gluteal incision	8	none
7	Rothchild [11]	1969	65	F	17.4	L	left ureter	open, lateral peritoneum was brought beneath the ureter	NA	none
8	Franken [12]	1969	58	F	none	B	bilateral ureter	open, repair	NA	none
9	Ghahremani [15]	1991	72	F	none	R	ileum	transgluteal approach	NA	none
10	Ivanov [17]	1993	60	F	none	R	cecum, appendix, small bowel and sigmoid colon	Open	5	none
11	Epner [18]	1994	86	F	none	L	left ureter	Antibiotics	NA	NA
12	Ritschel [19]	1995	51	F	13.2	L	left ureter	double J stent→ fail, open	NA	none
13	Losanoff [20]	1995	29	F	none	R	terminal ileum	transgluteal approach	4	none
14	Hayashi [21]	1995	44	F	none	L	ileum and urinary bladder	open and transgluteal approach	6	none
15	Servant [22]	1998	66	F	none	L	ileum and rectosigmoid colon	Open	NA	perforation before operation
16	Gee [24]	1999	60	F	25.0	L	left ureter	Laparoscopy	24	none
17	Noller [25]	2000	62	F	none	L	left ureter	left Gibson incision, retroperitoneal approach	NA	none
18	Yu [26]	2002	71	F	none	R	ileum	open, resection of the ileum	12	none
19	Touloupidis [27]	2006	61	F	none	R	right ureter	open, ureterolysis, reimplantation of the ureter in psoas hitch	NA	NA
20	Kohashi [28]	2006	80	F	none	R	small bowel	open	13	none
21	Dundamadappa [29]	2006	90	F	none	R	right ovary	open	NA	NA
22	Skipworth [30]	2006	36	F	none	R	liposarcoma	abdomino-perineal approach	24	none
23	Witney Smith [31]	2007	59	F	none	L	left ureter	laparoscopy	3	none
24	Loffroy [32]	2007	81	F	none	L	left ureter	open, resection of ureter, doubleJstent	3	none
25	Tsai [33]	2008	91	F	none	L	left ureter	observation	NA	none
26	Tokunaga [34]	2008	72	F	none	R	small bowel	transgluteal approach	NA	none
27	Speeg [35]	2009	82	F	none	L	left ureter	laparoscopy→open,resection of ureter	1.5	none
28	Paira [36]	2010	35	F	none	L	dermoid cyst	laparoscopy→open,resection of tumor	NA	NA
29	Clemens [37]	2010	80	F	none	L	left ureter	double J stent	NA	none
30	Chitranjan [38]	2010	55	F	none	L	sigmoid colon	NA	NA	NA
31	Bernard [39]	2010	72	F	none	R	small bowel, right ovary	laparoscopy	12	none
32	Singh [40]	2011	79	F	none	R	preperitoneal fat	robot	NA	none
33	Rather [41]	2011	80	F	none	L	small bowel	open	7	none
34	Sugimoto [42]	2011	76	F	none	L	left ureter	stent	3	none
35	Lopez [43]	2012	50	F	none	R	lipoma	transgluteal approach, resection	6	none
36	Andraus [44]	2012	64	F	none	L	ascites	NA	none	death
37	Whybum [45]	2013	74	F	none	B	bilateral ureter	laparoscopy	NA	NA
38	Labib [46]	2013	80	F	none	R	colon	observation	NA	NA
39	Pimenta [48]	2014	55	F	none	L	lipoma	transgluteal approach	24	none
40	Tsuzaka [49]	2014	78	F	14.5	L	left ureter	laparoscopy	8	none
41	Kato [50]	2014	72	F	none	L	left ureter	stent	72	recurrence after stent removal
42	Duran [51]	2015	39	F	none	L	osteolipoma	surgery (no data in detail)	NA	NA
43	Salari [52]	2015	87	F	14.4	R	right ureter	stent	12	none
44	Yanagi [53]	2015	92	F	none	L	left ureter	stent	12	none
45	Dulskas [54]	2015	53	M	29.0	R	lipoma	open, resection	NA	none
46	Colombo [56]	2016	65	F	21.0	R	right ovary, adnexa	laparoscopy	3	none
47	Regelman [57]	2016	60	F	none	L	left ureter	robotic, ureterolysis	6	none
48	Demetriou [58]	2016	76	F	none	L	left ureter	observation	6	none
49	Imamura [59]	2006	83	F	14.0	R	ileum	open	1	pneumonia
50	Uchida [60]	2010	75	F	14.1	R	ileum	observation	15	none
51	Tanaka [61]	2011	84	F	12.4	R	appendix	open, ileocecectomy	6	anastomotic leakage
52	Ema [62]	2011	75	F	16.4	L	ileum	open, ileocecectomy	20	none
53	Eriguchi [63]	2012	74	M	17.2	L	left ureter	DJ stent	NA	none
54	Asanuma [64]	2012	83	F	19.6	R	small bowel	open	7	none
55	Tsutsui [65]	2014	76	F	21.2	L	left ureter	DJ stent	6	none
56	Taguchi [66]	2014	84	F	22.9	R	right ovary	lap, resection of right ovary and right adnexa uteri	12	none
57	Iida [67]	2015	60	F	17.1	R	right ovary and right fimbriae of uterine tube	lap, patch closure	12	none
58	Kise [68]	2016	36	F	17.5	L	left ureter	DJstent	12	none
59	Nitta [69]	2016	93	F	19.2	R	small bowel	open, resection of paro-ovarian cyst	NA	recurrence
60	Ishikawa [70]	2017	77	F	23.2	R	small bowel	lap, mesh plug and patch	12	none
61	Our patient	2018	86	F	20.9	L	left ureter	manual reduction	10	none

*NA*=not applicable, BMI = body mass index, L = Left, R = Right, M = Male, F = Female.

**Table 2:** Reports of child patients with a sciatic hernia.

No.	Author	Year	Age (days)	M/F	BMI (kg/m^2^)	L/R	Contents	Treatment	Follow- up (m)	Complications
1	Henegar [5]	1952	660	M	none	R	cecum and right ureter	right gluteal incision	6	hypertrophy of the scar
2	Gaffney [7]	1958	150	F	none	L	none	left gluteal incision	12	none
3	Chamberlain [8]	1958	2	F	none	L	retroperitoneal teratoma	open, resection of the tumor	none	death due to bronchopneumonia
4	Franken [12]	1969	60	M	none	B	bilateral ureter	none	NA	NA
5	Franken [12]	1969	35	F	none	L	rectosigmoid	none, spontaneous recovery	NA	NA
6	Bohnert [13]	1971	60	M	none	B	bilateral ureter	none	12	Urinary Tract Infection
7	Lebowit [14]	1973	45	F	none	R	right ureter	none	NA	NA
8	Attar [16]	1992	540	M	none	R	sigmoid colon	right gluteal incision	NA	NA
9	Arat [23]	1998	90	F	none	L	left ureter	NA	NA	NA
10	Seifarth [47]	2014	49	M	none	R	ileum	laparoscopy→open	36	none
11	Nosek [55]	2015	1	M	none	R	duplication of rectum	laparoscopy→transgluteal, endorectal pull through	24	none

*NA*=not applicable, BMI = Body mass index, L = left, R = right, M = Male, F = Female.

Of 61 adults including the present patient, 57 (93%) were female. Of the 11 children found in this review, five (45%) were female. This suggests that sciatic hernias tend to occur more frequently in adult females. However, there is no difference in incidence between genders in children. Atrophy of the piriformis muscle has been described as a predisposing factor. Therefore, elderly patients with decreased body mass index tend to have this condition. Common symptoms include unilateral lower abdominal pain, lumbar pain, and bulging of one buttock. In adults, the hernia contents have been reported to include the ureter (N = 26), small bowel (N = 14), tumors (myxoma, lipoma, osteolipoma, liposarcoma, dermoid cyst) (N = 8), colon (N = 2), ovary (N = 2), appendix (N = 1), ascites (N = 1), preperitoneal fat (N = 1), multiple organs (N = 6).

Formerly, the diagnosis of sciatic hernia was made by physical examination (e.g. bulging) or at the time of operation. After the advent of the CT scan, it is the mainstay of diagnostic modalities to identify a sciatic hernia. Intravenous pyelogram or retrograde pyelogram have been performed for some patients with ureterosciatic hernias. The “curlicue” sign of the ureter was specific for this entity if the hernia contains the ureter [6].

The treatment of a patient with a sciatic hernia depends on the hernia contents and commonly includes surgery (usually, open repair or transgluteal repair) or placement of a ureteral stent if the ureter is involved. Open reduction with laparotomy was performed in 24 patients in the series reviewed. Recently, nine patients were reportedly treated laparoscopically and two by robotic-assisted surgery. Two patients underwent conversion from laparoscopy to laparotomy. There are no reports of successful transvaginal closed manual reduction.

Transvaginal closed manual reduction was used to treat the present patient. With the patient in the prone position, the assistant places the ultrasound probe on the left buttock. The exact location of the hernia was confirmed by the CT scan. After confirming the location of the hernia, the operator inserted the right hand into the vagina, while extending the index and middle fingers (Fig. 3). The entire hand of the examiner should be inserted into the vagina. The index and middle fingers were positioned at the posterior fornix of the vagina, and traction applied with the fingertips in a repetitive manner, reducing the invaginated left ureter. The ureter was reduced along with adjacent connective tissue. After that, the operator and assistant confirmed reduction with ultrasound imaging.

In the combined series of 72 patients, postoperative complications include one death from sepsis, one anastomotic leak, one patient developed heart failure, one patient developed pneumonia, and two recurrences occurred in adults. In children, there was one death from bronchopneumonia. Two recurrences are reported after a repair without using mesh (1/20) and after removal of the ureteral stent (1/3). There are deaths reported after operative repair.

Transvaginal manual reduction is less invasive and easier than other reported approaches. If there are no suspicion of strangulation of the invaginated tissue, it may be considered as the first modality to be used. However, there is a possibility of recurrence because the hernia defect has not been definitively closed. In addition, this maneuver is not applicable to men, children (female children have an intact hymen and small vagina), and possibly, young females whose vagina may not be able to accommodate the examiner's hand.

## 4 Conclusion

An incarcerated sciatic hernia in women can be manually reduced. To determine the best management strategy, further studies and collection of data regarding this rare entity, treatment and follow-up are necessary.

## Conflicts of interest

All authors have no conflict of interest.

## Sources of funding

Authors had no sources of funding.

## Ethical approval

IRB/Ethics Committee ruled that approval was not required for this study.

## Consent

Written informed consent was obtained from the patients for publication of this case report and accompanying images. A copy of the written consent is available for review by the Editor-in-Chief of this journal on request.

## Author contribution

The work presented was carried out in collaboration between all authors. JK, KY, TS, AKL, and TK defined the research theme, discussed analyses and approved the final version to be published. JK analyzed the data, interpreted the results and wrote the paper.

## Registration of research studies

There is no need to register because it is a case report.

## Guarantor

Jiro Kimura.

## Provenance and peer review

Not commissioned, externally peer-reviewed.

## References

[1] Summers J.E. (1922). Sciatic hernia: report of a case complicated with myxomatous tumor of the scrotum. Ann. Surg..

[2] Agha R.A., Borrelli M.R., Farwana R., Koshy K., Fowler A., Orgill D.P., For the SCARE Group (2018). The SCARE 2018 statement: updating consensus Surgical CAse REport (SCARE) guidelines. Int. J. Surg..

[3] Lindbom A. (1947). Unusual ureteral obstruction by herniation of ureter into sciatic foramen. Acta Radiol..

[4] Lawson R. (1948). Sciatic hernia. Can. Med. Assoc. J..

[5] Henegar G.C., Hudson C.B., Jensen G.L. (1952). Sciatic notch hernia; report of a case and description of a new operative approach. AMA Arch. Surg..

[6] Beck W.C., Baurys W., Brochu J., Morton W.A. (1952). Herniation of the ureter into the sciatic foramen (“curlicue ureter”). J. Am. Med. Assoc..

[7] Gaffney L.B., Schanno J.F. (1958). Sciatic hernia; a case of congenital occurrence. Am. J. Surg..

[8] Chamberlain W.H., Motsay D.S., Barone A.C. (1958). An unusual sciatic hernia in a deceitful masquerade. Guthrie Clin. Bull..

[9] Kerry R.L., Tygart R.L., Glas W.W. (1964). Lipoma: a “reversed” perineal sciatic hernia. Am. J. Surg..

[10] Sadek H.M., Kiss D.R., Vasconcelos E. (1970). Sciatic hernia caused by a neurofibroma. Surgical repair with a stainless wire mesh. Int. Surg..

[11] Rothchild T.P. (1969). Ureteral hernia. Report of a case of herniation of the ureter into the sciatic foramen. Arch. Surg..

[12] Franken E.A., Smith E.E. (1969). Sciatic hernia: report of three cases including two with bilateral ureteral involvement. Am. J. Roentgenol. Radium Ther. Nucl. Med..

[13] Bohnert W.W. (1971). Ureteral sciatic hernias: case report of an infant with bilateral ureteral herniation into the sciatic foramina. J. Urol..

[14] Lebowitz R.L. (1973). Ureteral sciatic hernia. Pediatr. Radiol..

[15] Ghahremani G.G., Michael A.S. (1991). Sciatic hernia with incarcerated ileum: CT and radiographic diagnosis. Gastrointest. Radiol..

[16] Attah M., Jibril J.A., Yakubu A., Kalayi G.D., Nmadu P.T. (1992). Congenital sciatic hernia. J. Pediatr. Surg..

[17] Ivanov N.T., Losanoff J.E., Kjossev K.T. (1994). Recurrent sciatic hernia treated by prosthetic mesh reinforcement of the pelvic floor. Br. J. Surg..

[18] Epner S.L., Lautin E.M. (1994). Case report: intermittent sciatic herniation of the ureter. Clin. Radiol..

[19] Ritschel S., Heimbach D., Schoeneich G. (1996). Ureterosciatic hernia. Scand. J. Urol. Nephrol..

[20] Losanoff J., Kjossev K. (1995). Sciatic hernia. Acta Chir. Belg..

[21] Hayashi N., Suwa T., Kimura F., Okuno A., Ishizuka M., Kakizaki S. (1995). Radiographic diagnosis and surgical repair of a sciatic hernia: report of a case. Surg. Today.

[22] Servant C.T. (1998). An unusual cause of sciatica. A case report. Spine (Phila. Pa. 1976).

[23] Arat A., Haliloglu M. (1998). Ureteral-sciatic hernia in a child demonstrated by voiding cystography. J. Urol..

[24] Gee J., Munson J.L., Smith J.J. (1999). Laparoscopic repair of ureterosciatic hernia. Urology.

[25] Noller M.W., Noller D.W. (2000). Ureteral sciatic hernia demonstrated on retrograde urography and surgically repaired with Boari flap technique. J. Urol..

[26] Yu P.C., Ko S.F., Lee T.Y., Ng S.H., Huang C.C., Wan Y.L. (2002). Small bowel obstruction due to incarcerated sciatic hernia: ultrasound diagnosis. Br. J. Radiol..

[27] Touloupidis S., Kalaitzis C., Schneider A., Patris E., Kolias A. (2006). Ureterosciatic hernia with compression of the sciatic nerve. Int. Urol. Nephrol..

[28] Kohashi T., Itamoto T., Yamasaki H., Yokoya H., Yonehara S., Asahara T. (2006). Sciatic hernia with an early-stage adenocarcinoma of the appendix: report of a case. Hiroshima J. Med. Sci..

[29] Dundamadappa S.K., Tsou I.Y., Goh J.S. (2006). Clinics in diagnostic imaging (107). Singap. Med. J..

[30] Skipworth R.J., Smith G.H., Stewart K.J., Anderson D.N. (2006). The tip of the iceberg: a giant pelvic atypical lipoma presenting as a sciatic hernia. World J. Surg. Oncol..

[31] Witney-Smith C., Undre S., Salter V., Al-Akraa M. (2007). An unusual case of a ureteric hernia into the sciatic foramen causing urinary sepsis: successfully treated laparoscopically. Ann. R. Coll. Surg. Engl..

[32] Loffroy R., Bry J., Guiu B., Dubruille T., Michel F., Cercueil J.P. (2007). Ureterosciatic hernia: a rare cause of ureteral obstruction visualized by multislice helical computed tomography. Urology.

[33] Tsai P.J., Lin J.T., Wu T.T., Tsai C.C. (2008). Ureterosciatic hernia causes obstructive uropathy. J. Chin. Med. Assoc..

[34] Tokunaga M., Shirabe K., Yamashita N., Hiki N., Yamaguchi T. (2008). Bowel obstruction due to sciatic hernia. Dig. Surg..

[35] Speeg J.S., Vanlangendonck R.M., Fusilier H., Richardson W.S. (2009). An unusual presentation of a sciatic hernia. Am. Surg..

[36] Paira S.K., Nath S., Mukherjee R., Chaudhary T., Bandopadhyay S.K., Ghosh P.S. (2010). Herniation of a broad ligament dermoid cyst through sciatic foramen—a rare cause of gluteal swelling. J. Indian Med. Assoc..

[37] Clemens A.J., Thiel D.D., Broderick G.A. (2010). Ureterosciatic hernia. J. Urol..

[38] Chitranjan Kandpal H., Madhusudhan K.S. (2010). Sciatic hernia causing sciatica: MRI and MR neurography showing entrapment of sciatic nerve. Br. J. Radiol..

[39] Bernard A.C., Lee C., Hoskins J., Lee J., Patel S., Ginn G. (2010). Sciatic hernia: laparoscopic transabdominal extraperitoneal repair with plug and patch. Hernia.

[40] Singh I., Hudson J.E., Richards K.A., Hemal A.K. (2011). Robot assisted laparoscopic repair of sciatic hernia (RASH): a case report. Indian J. Surg..

[41] Rather S.A., Dar T.I., Malik A.A., Parray F.Q., Ahmad M., Asrar S. (2011). Sciatic hernia clinically mimicking obturator hernia, missed by ultrasonography: case report. Ulus. Travma Acil Cerrahi Derg..

[42] Sugimoto M., Iwai H., Kobayashi T., Morokuma F., Kanou T., Tokuda N. (2011). Ureterosciatic hernia successfully treated by ureteral stent placement. Int. J. Urol..

[43] López-Tomassetti Fernández E.M., Hernández J.R., Esparragon J.C., García A.T., Jorge V.N. (2012). Intermuscular lipoma of the gluteus muscles compressing the sciatic nerve: an inverted sciatic hernia. J. Neurosurg..

[44] Andraus W., Haddad L.B., Ferro O.C., D’Albuquerque L.A. (2012). Sciatic hernia mimicking perianal abscess in a cirrhotic patient. Case Rep. Med..

[45] Whyburn J.J., Alizadeh A. (2013). Acute renal failure caused by bilateral ureteral herniation through the sciatic foramen. Urology.

[46] Labib P.L., Malik S.N. (2013). Choice of imaging modality in the diagnosis of sciatic hernia. J. Surg. Case Rep..

[47] Seifarth F.G., Kundu N., Magnuson D.K. (2014). Congenital sciatic hernia. Pediatr. Surg. Int..

[48] Pimenta R., Matos R.M., Proença R., Pereira H.R., Pinto R. (2014). Giant buttock lipoma with an atypical presentation as a sciatic hernia—case report. Acta Reumatol. Port..

[49] Tsuzaka Y., Saisu K., Tsuru N., Homma Y., Ihara H. (2014). Laparoscopic repair of a ureteric sciatic hernia: report of a case. Case Rep. Urol..

[50] Kato T., Komiya A., Ikeda R., Nakamura T., Akakura K. (2014). Minimally invasive endourological techniques may provide a novel method for relieving urinary obstruction due to ureterosciatic herniation. Case Rep. Nephrol. Dial..

[51] Duran S., Cavusoglu M., Elverici E., Unal T.D. (2015). A giant retroperitoneal lipoma presenting as a sciatic hernia: MRI findings. JBR-BTR.

[52] Salari K., Yura E.M., Harisinghani M., Eisner B.H. (2015). Evaluation and treatment of a ureterosciatic hernia causing hydronephrosis and renal colic. J. Endourol. Case Rep..

[53] Yanagi K., Kan A., Sejima T., Takenaka A. (2015). Treatment of ureterosciatic hernia with a ureteral stent. Case Rep. Nephrol. Dial..

[54] Dulskas A., Poskus E., Jurevicius S., Strupas K. (2015). Giant gluteal lipoma presenting as a sciatic hernia. Hernia.

[55] Nosek M., Golonka A., Kalińska-Lipert A., Nachulewicz P. (2015). Rectal duplication with sciatic hernia. Wideochir. Inne Tech. Maloinwazyjne.

[56] Colombo F., Calcagno P., Crespi M., Bonzanini O., Sampietro G.M., Foschi D. (2017). Laparoscopic repair of a sciatic hernia containing the ipsilateral ovary: case report and review of the literature. J. Laparoendosc. Adv. Surg. Tech. A.

[57] Regelman M., Raman J.D. (2016). Robotic assisted laparoscopic repair of a symptomatic ureterosciatic hernia. Can. J. Urol..

[58] Demetriou G.A., Perera S., Halkias C., Ahmed S. (2016). Seventy-six-year-old woman with an unusual anatomy of the left ureter. BMJ Case Rep..

[59] Imamura N., Shimayama T., Sueta H., Kawano K., Haruyama Y., Chijiiwa K. (2006). A case of sciatic hernia. Jpn. J. Gastroenterol. Surg..

[60] Uchida H., Kawasaki H., Nishida T., Umemoto K., Miyoshi K., Inada Y. (2010). A case of ileus due to sciatic hernia treated with conservative therapy. Jpn. J. Gastroenterol. Surg..

[61] Tanaka R., Suzuki S., Okada T., Aono T. (2011). A case report of sciatic hernia with an abscess due to perforation of the appendix. J. Jpn. Surg. Assoc..

[62] Ema A., Koyanagi K., Nakagawa M., Asagoe T., Matsumoto K., Nagase T. (2011). A case of sciatic hernia incarcerated through lesser sciatic foramen. Jpn. J. Gastroenterol. Surg..

[63] Eriguchi T., Yoshizawa R., Tamura M., Namiki S., Numata I., Tsuboi M. (2012). A case of ureterosciatic hernia, successfully treated with ureteral catheter. Jpn. J. Urol. Surg..

[64] Asanuma S., Terasaki M., Okamoto Y., Tanaka K., Suzumura K., Kamiya T. (2012). A case of bowel obstruction due to an incarcerated sciatic hernia. J. Jpn. Surg. Assoc..

[65] Tsutsui A., Shirouzu T., Naganuma H., Iwai H., Harano M. (2014). Ureterosciatic hernia treated by ureteral stent: a case report. Nishinihon J. Urol..

[66] Taguchi Y., Isaji T., Sakamoto E., Komatsu S., Norimiizu S., Singu Y. (2014). A case of sciatic hernia repair by a laparoscopic approach. J. Jpn. Surg. Assoc..

[67] Iida M., Ueno T., Maeda Y., Hazama S., Nagano H. (2015). A case of sciatic hernia treated with laparoscopic repair. J. Jpn. Hernia Soc..

[68] Kise H., Tachino H., Hotta Y. (2016). Ureterosciatic hernia treated with ureteral stent: a case report. Nishinihon J. Urol..

[69] Nitta M., Shimada H., Nishi T., Miyakita H., Ozawa S., Makuuchi Y. (2016). A case of sciatic hernia coexisting with an obturator hernia. J. Jpn. Surg. Assoc..

[70] Ishikawa M., Iwamoto H., Kondo S., Sasamoto A., Mori M., Miyamoto K. (2017). A case of sciatic hernia repair by a laparoscopic approach. J. Jpn. Surg. Assoc..

